# Patient experiences of resection versus responsive neurostimulation for drug-resistant epilepsy

**DOI:** 10.1016/j.yebeh.2024.109707

**Published:** 2024-03-01

**Authors:** Tobias Haeusermann, Emily Yang Liu, Kristina Celeste Fong, Daniel Dohan, Winston Chiong

**Affiliations:** a UCSF Weill Institute for Neurosciences, United States; b UCSF Bioethics, United States; c UCSF Epilepsy Center, United States; d UCSF Institute for Health Policy Studies, United States

**Keywords:** Drug-resistant epilepsy, Surgical resection, Responsive neurostimulation, Patient experience

## Abstract

This study explored illness experiences and decision-making among patients with epilepsy who underwent two different types of surgical interventions: resection versus implantation of the NeuroPace Responsive Neurostimulation System (RNS). We recruited 31 participants from a level four epilepsy center in an academic medical institution. We observed 22 patient clinic visits (resection: n = 10, RNS: n = 12) and conducted 18 in-depth patient interviews (resection: n = seven, RNS: n = 11); most visits and interviews included patient caregivers. Using an applied ethnographic approach, we identified three major themes in the experiences of resection versus RNS patients. First, for patients in both cohorts, the therapeutic journey was circuitous in ways that defied standardized first-, second-, and third- line of care models. Second, in conceptualizing risk, resection patients emphasized the permanent loss of “taking out” brain tissue whereas RNS patients highlighted the reversibility of “putting in“ a device. Lastly, in considering benefit, resection patients perceived their surgery as potentially curative while RNS patients understood implantation as primarily palliative with possible additional diagnostic benefit from chronic electrocorticography. Insight into the perspectives of patients and caregivers may help identify key topics for counseling and exploration by clinicians.

## 1. Introduction

Despite advances in anti-seizure medications, the proportion of patients with drug-resistant epilepsy (DRE) remains unchanged at one-third of all cases [1,2]. However, <1 % of patients with DRE are referred to tertiary epilepsy centers, and of these, only 10–30 % ultimately pursue surgery, rendering epilepsy surgery, “one of the most underutilized of all accepted medical treatments in the world” [3–5].

In addition to socioeconomic, resource, and knowledge barriers impeding access to care, patient attitudes and perspectives related to epilepsy surgery may further impact their willingness to pursue surgical evaluations [4]. For patients, this results in increased risk of morbidity and mortality through Sudden Unexpected Death in Epilepsy (SUDEP), injuries related to seizures, significant disability, poor psychosocial health, and overall diminished quality of life [6,7]. Clinicians of varying experience and expertise may be unsure how best to counsel patients and caregivers, given the prognostic uncertainty and technical complexity of surgical interventions for epilepsy. Here, effective counseling may be bolstered by deep insight into in patient and caregiver perspectives during and after the surgical evaluation process [8]. However, we have limited data into patient and caregiver experiences during the surgical evaluation process [8–12].

We seek to address this gap in the literature by providing preliminary insight into the perspectives and experiences of two patient cohorts with DRE. One cohort underwent surgical resection, whereas the other was implanted with the NeuroPace Responsive Neurostimulation System (RNS) [13,14]. These two surgical options encompass many of the nuanced considerations involved in the surgical management of epilepsy. Within the literature, such considerations include (1) impact on self-perception, personality traits, and essential functions with resection and neuromodulation; (2) issues of patient privacy, access to healthcare data, and proprietary interests with chronic electrocorticography (ECoG); (3) safety concerns related to novel neurodevices; and (4) shifts in patient fears and expectations related to treatment outcomes and physician engagement in the post-operative setting [15]. Additionally, novel neurodevices like RNS may tap into public anxieties related to emerging brain-computer interface technology [16]. Our study focuses on the real-world experiences and lived implications of these therapies for patients and caregivers, to inform and support clinician counseling.

## 2. Methods

### 2.1. Study design and rationale

This study is part of a larger multi-arm project examining the lived experiences of key stakeholders at the forefront of emerging therapeutic neuromodulatory devices, of which RNS is a prototype [17]. Here, we specifically sought to explore the perspectives and experiences of both patients undergoing traditional resection as well as those implanted with RNS to capture the spectrum of considerations in surgical epilepsy more fully. We used an applied ethnographic approach that combines grounded observation with semi-structured interviews [18–20].

Interest in qualitative methodologies within epilepsy and health sciences research is growing. This in part reflects an increasing awareness of the limitations of quantitative, often experiment-based, approaches in studying complex, embedded phenomena. Whereas quantitative approaches seek to establish causal relationships, test hypotheses, and define representative characteristics of large populations, qualitative approaches aim to explore singular, multivariable phenomena in natural – as opposed to experimental – environments [21,22]. In their 2015 epilepsy research review, Rapport et al. identified patient and caregiver experiences during the surgical evaluation process as an area particularly well-suited to a qualitative methodology [22].

### 2.2. Recruitment

We applied a cross-sectional design with purposeful sampling, enrolling: (1) individuals being treated for DRE; who are (2) candidates for or have undergone resection and/or RNS; (3) age ≥ 18 years at the time of surgery; (4) able to participate meaningfully in in-depth, ethnographic interviews; and (5) fluent in English. Recruitment occurred between 2018 and 2019 from a single level four epilepsy center in an academic medical institution [23]. To identify eligible patients, the study team observed the center’s biweekly epilepsy surgical case conference, after which the treating epileptologist was contacted to confirm a given patient’s eligibility. At the time of data collection, all our patients had been designated either as a resection or RNS candidate by the clinical team. Fieldwork began in June 2018 and continued through June 2020, in parallel with recruitment. Two overt clinic observations were conducted for each patient – one pre-operatively and one post-operatively. Semi-structured interviews continued through May 2022, with one to two interviews conducted with each patient participant and, when available, the patient’s primary caregiver in the post-operative setting.

The study team first approached patients at a scheduled medical appointment, during which the epileptologist sought verbal permission for the researcher to observe the appointment. At the end of this first observed visit, patients and caregivers were invited by the study team to participate in follow-up interviews. Those interested provided their contact information. Interviews were conducted either in person or virtually on Zoom, in compliance with COVID-19 restrictions and per participant preference. Prior to each interview, written informed consent was obtained in person or via DocuSign. Three non-clinical study members performed all clinic observations and semi-structured interviewers.

### 2.3. Participants

A total of 31 participants were initially recruited for the grounded observation phase of the study. Two participants did not proceed to surgical treatment; one was excluded because they opted for VNS; five were either lost to follow-up or withdrew due to non-medical reasons; and one participant did not return the written consent form. The data from these participants were excluded from the study. Of the remaining 22 participants, 18 proceeded to follow-up interviews. Of these, 11 were RNS patients, including one that later had a resection and another that underwent a radiofrequency ablation based on chronic ECoG data from RNS; seven were resection patients. Caregivers accompanying patients to their appointments were also invited to participate in interviews. 14 caregivers, nine from the RNS cohort and five from the resection cohort, were interviewed. Demographic characteristics on race and ethnicity were systematically derived from electronic health records and are provided separately here. One participant was identified as Asian; one as African-American/Black; 14 as White; and six as Other. For ethnicity, five were identified as Hispanic or Latino. Other characteristics are presented in Table 1.

### 2.4. Data collection

#### Clinic observations:

Ethnographers applied established fieldwork methods to document behaviors and interactions of patients, caregivers, and clinicians. To minimize disruption while enhancing later recollection, researchers collected abbreviated in situ field notes on paper [24]. Upon leaving the field site, observers transcribed notes via word processing software into full ethnographic reports with “thick descriptions” that contained the context and interpretations of observed behaviors and interactions [25].

#### Follow-up interviews:

Interviews were conducted either in person outside the clinical site or via Zoom. Interview questions broadly explored four key areas, with additional time allotted for emergent themes: (1) Discussion of Observed Visit, (2) Illness and Treatment Experiences, (3) Fears and Hopes for the Future, and (4) Personal Background. An interview guide is attached in the Appendix. With permission, interviews were audio-recorded for later transcription, and the researchers wrote memos to monitor reflexivity during the data collection process.

### 2.5. Data analysis

Observational field notes and transcribed interviews were imported into Atlas.ti software. The study team generated an initial codebook based on broad, pre-determined categories, such as experience of epilepsy, fears and hopes related to surgery, etc. During analysis, we created additional, inductive codes to note concepts that emerged during data review. For rigor, two study members doubled-coded two sets of fieldnotes and transcripts, subsequently comparing codes and resolving discrepancies through deliberation [26]. A single study member then coded all remaining fieldnotes and transcripts.

### 2.6. Ethics

The study was approved by the Committee on Human Research at our home institution. Informed consent was collected prior to data collection, and participants were free to decline to answer interview questions, stop the interview, or withdraw from the study altogether at any point. In accordance with our approved protocol, in this manuscript, we refer to participants using coded identifiers and do not identify the research site. Transcribed interviews and observation notes were de-identified and stored in a secure cloud-based file collaboration tool, managed, and supported by the Information Technology Services at our home institution.

## 3. Results

Three key themes emerged in our analysis of clinic observations and semi-structured interviews. First, for patients in both resection and RNS cohorts, the therapeutic journey was circuitous in ways that defied standardized first-, second-, and third- lines of care models. During observed visits, clinicians discussed either resection or RNS with patients and caregivers based on the group consensus from surgical case conference, with little reference to other surgical options. Despite this, participants described these therapies in opposing terms. For example, resection patients described an experience of “taking out” whereas RNS patients depicted one of “putting in,” with the latter understood as a reversible process. Similarly, resection patients perceived their surgery as potentially curative while RNS patients understood implantation as primarily palliative, though with possible additional diagnostic benefit from chronic ECoG.

### 3.1. Finding 1 – Circuitous journeys

Line-of-care treatments models in epilepsy present medications as first-line, ablation and resection as second-line, and neurodevices such as RNS as third-line [17]. However, our participants rarely progressed along a sequential therapeutic trajectory.

First, the decision to undergo epilepsy surgery is a high-stakes one, often preceded by years of attempts at pharmacologic management. For several participants, previous misdiagnoses and ineffective medication trials had left them disheartened and skeptical of achieving positive future outcomes. Generally, patients had been referred to the epilepsy center by a neurologist, whereupon their seizures – etiology, onset, semiology, etc. – were investigated via a comprehensive phase I evaluation. The sporadic nature of seizures sometimes necessitated long periods of observation. Only once the clinical and electrographic seizure profile was determined were patients presented with surgical options. Regularly, participants and caregivers described their journeys as “winding,” “confusing,” and “chaotic.”

However, even following epilepsy surgery, patients’ lived experiences did not always follow projected treatment pathways. For some resection patients, seizures would evolve in unexpected and debilitating ways post-resection, necessitating ongoing trials of medication, complementary therapies such as the modified Atkins or ketogenic diet, or at times even a redo operation. Most RNS participants in our study proceeded via the expected pathway of device implantation and activation with subsequent iterative programming of the device for improved seizure detection and treatment. However, one participant unexpectedly achieved seizure freedom following RNS implantation, and the device’s stimulation function was never activated. For two participants, RNS stimulation did not lead to satisfactory seizure reduction, but continual ECoG enabled the detection of asingle seizure focus and subsequent resective surgery. Indeed, the ability for chronic ECoG complicates standard line-of-care pathways by creating a potential “reverse” pathway from third- to second- lines of care.

Oftentimes, patients were left feeling uncertain and nervous about the possible different complex paths their epilepsy could follow post-operatively. One resection participant ruefully noted, “If I had a seizure today… that comes with huge weight and consequences. It’s like, gosh, was this worth it? […]How is life going to change? I mean in all honesty, I do have that fear” (Resection participant 1212).

### 3.2. Finding 2 – Loss versus reversibility

Participants in both cohorts expressed fears of post-operative complications and negative outcomes. In particular, resection and RNS patients emphasized the irreverisiblity versus reversibility of these procedures in theirrisk assessments, related to the potential for permanent loss of essential functions and personality traits.

In one illustrative example, a RNS participant, who had previously undergone resection, compared the two as follows:

“…with the RNS device they were putting something in. You know, there were wires that were going to my brain to stimulate it, they weren’t cutting something out of my head. So, that’s the difference between the RNS and the procedure. […] I was excited…I wasn’t scared at all. The last surgery I was scared” (RNS participant 1111).

In other interviews, participants emphasized concerns primarily related to the irreversibility of resection. One woman (RNS participant 1115) who had RNS placed followed by a targeted laser ablation explained her initial trepidation regarding resection as, “if you’re removing part of the brain…if something goes wrong, [you] can’t put it back.” Similarly, another participant in his 20s (Resection participant 1203) with DRE with focal left posterior temporal onset explained “… when they told me…it was right on the area of comprehension and speech [and] there’s a possibility I won’t be able to speak again, that’s a big thing.”

In contrast, RNS was perceived as a safer alternative. One participant (RNS participant 1115) explained this was because “[the] device could be removed, and nothing would truly change.” Another participant (RNS participant 1113) further emphasized “It was pretty minor on the level of brain surgery. It was just putting electrodes into my brain.”

### 3.3. Finding 3 - curative versus palliative

Participants undergoing resection versus RNS applied different metrics for evaluating the success or failure of treatment, based around notions of cure.

Resection is understood as a potentially curative therapy. In our study, resection participants commonly framed surgical outcomes in “all-or-nothing” terms. In the post-resection period, patients often dwelled on the possibility of latent seizures, with many defining breakthrough seizures as a failure of surgical resection even if there was an overall reduction in seizure burden. Here, observation notes from a post-operative visit for one resection patient (Resection participation 1208) illustrate the devasting impact the return of seizures can have.

The caregiver immediately sobs and covers her hands with her face. The epileptologist shuffles toward the caregiver and grabs a box of tissues. The caregiver takes a few tissues and wipes her tears. The epileptologist correctly recognizes this…as a reaction to the return of the patient’s seizures and asks when the seizures began. The caregiver struggles to speak but manages to explain that on [redacted date], the patient told her that he thought he had a nocturnal seizure, but the caregiver thought, “That can’t be.” The patient had been seizure-free since the operation, and the caregiver thought maybe the patient was imagining the seizure or over-thinking. […] The caregiver says that post-resection has “been a huge improvement, but I was just so sure. It’s really devastating” (Resection participant 1208).

In contrast, RNS is not presented as a curative therapy, and patients are generally counseled to not expect immediate and permanent seizure freedom. Instead, the device’s positive effects occur over time, with iterative improvements in seizure detection and stimulation parameters. In our study, RNS patients did not see treatment outcomes in “all-or-nothing” terms. For example, one caregiver of an RNS reported:

“One of the major goals going into the device was just reducing the amount of seizures…and then with better seizure control just having her be a little bit more independent.…Before, once she had a seizure, she’d have to lay down, and it would take her a while to kind of regroup. And now she can have a small seizure and then just kind of pick up, you know, where she was” (Caregiver of RNS participant 1104).

Similarly, another RNS patient felt that “being they were petit mal seizures, clearly that was a success, being that they could have been grand mal seizures” (RNS participant 1112).

Interestingly, there was a strong emphasis on increased therapeutic engagement as a post-surgical expectation that was unique to RNS patients, given the need for regular visits for review of data and device programming. One RNS participant (1113) shared, “It’s like I do all this work - I have brain surgery, and I can’t see anything about the impact. And that, to me, is really upsetting. And my doctor is wonderful. But she basically can’t tell me the data until my appointments every three months.”

## 4. Discussion

Patient perceptions of epilepsy surgery, expected treatment outcomes, and the relative risks of surgery versus that of uncontrolled seizures are significant factors in patient and caregiver decision-making for surgical epilepsy [9,10,27]. Patients undergoing surgical evaluation commonly identify their epileptologist as their primary source of information [10]. Our findings give preliminary insight into how patients and caregivers conceptualize the surgical evaluation process as well as the risks and benefits of epilepsy surgery. Based on this data, we have identified three potential domains for further exploration and counseling by clinicians, with specific recommendations outlined below.

1. The circuitous nature of the surgical process: Respondents in both groups reported complex diagnostic and treatment trajectories. Standard line-of-care treatment models impose a systematic framework for medical practice. For patients, however, diagnosis and treatment did not follow along such sequential steps. Our study participants shared experiences of an often circuitous, if not chaotic, process, spanning years to decades and several clinicians and health systems, from initial (re)diagnosis to multiple failed medication trials leading to an epilepsy center. Consequently, some may perceive referral to an epilepsy center as the “end” to their arduous journey, while others may fear that surgery marks the termination of the patient-physician relationship with their epileptologist. Early discussions exploring patient and caregiver experiences to date and their hopes for the future may provide an opportunity to explicit address the circuitous nature of the surgical process and help better temper unrealistic expectations and assuage fears of abandonment.
2. The perception of loss and reversibility: Resection patients emphasized the irreversibility of “taking out” and the risk of permanent loss of personality traits and essential functions. In contrast, RNS was understood in terms of gain with implantation of the device, potentially undermining patients’ risk estimates of RNS. Patients may benefit from a more extensive discussion of risk-mitigation strategies in the case of resection, specifically the incorporation of in-depth neuropsychological evaluation, Wada, advancing neuroimaging such as fMRI and MEG, and ultimately cortical mapping to guard against the removal of eloquent cortex [28]. Additionally, in certain cases, there may need to be a greater emphasis on the negative effects of uncontrolled seizures on behavior, cognition, and overall functional status [29]. Standard risks of implantation – bleeding, infection, and erosion – apply to RNS. Additionally, as a novel brain-computer interface, RNS is at the vanguard of emerging safety and ethical considerations. These range from issues of patient privacy with centralized, proprietary ownership of ECoG data to the potential impact of these devices on notions of self, embodiment, estrangement, and empowerment [15,17].
3. The dichotomy of curative and palliative: For many patients deemed eligible for resection, it was the first time in their epilepsy course they were presented with the prospect of a cure. The language of cure may heighten expectations, implying that the true indication of a successful resection is total seizure freedom. In contrast, RNS was discussed as a palliative therapy. The term “palliative” has strong associations with hospice and end-of-life care; it has since been adopted by other medical and surgical specialties but retains a connotation of “non-curative” [30]. Consequently, there may be an underestimation of the therapeutic potential of RNS by patients through termination of incipient seizures, positive effects of long-term neuromodulation, and the potential for identification of a single seizure focus for later resection through chronic ECoG. In both cases, the language of cure versus palliation may undermine opportunities for patients and clinicians to discuss realistic expectations for epilepsy surgery and explore patient-specific goals of treatments, such as elimination of convulsive seizures, regaining driving privileges, etc. Such language should be used with a sensitivity towards their embedded meanings by clinicians when counseling patients.

## 5. Limitations and next steps

Study participants were recruited from a single academic center and followed in the immediate *peri*-operative period. Consequently, this study was limited to a small sample size and is not fully representative of the population at large. Additionally, our findings only reflect patient and caregiver perspectives at the time of the study. However, the main aim of our study was not to provide representative or generalizable data. Rather, it was to give preliminary insights into patient and caregiver experiences during the surgical evaluation process, with the goal of informing and supporting clinician counseling and providing a framework for future studies. Another limitation is the potential impact of bias and subjectivity, which we counteracted through triangulation by having three study members perform clinic observations and then compare data with interview responses to improve validity and decreases retrospection bias. Furthermore, we attempted to standardize our coding process by double-coding an initial set of data and then adjudicating any discrepancies in the codebook.

Potential next steps include (1) reviewing data from observed visits to investigate various aspects of clinician counseling; (2) follow-up semi-structured interviews on the perceived impact of clinician counseling by patients and caregivers; (3) expanding to multiple study sites; and (4) exploring patient and caregiver perspectives beyond the immediate *peri*-operative period to see if and how these evolve over time.

## Supplementary Material

Interview Guide

## Figures and Tables

**Table 1:** Demographics of the final sample (n = 22).

Demographics	Number (%)/Mean (SD)	
Surgery type	RNS	Resection
Total participants	12 (55 %)	10 (45 %)
Male	3 (25 %)	7 (70 %)
Female	9 (75 %)	3 (30 %)
Age (as of 5/2022)	47 (13.6)	39 (11.5)
Month since surgery (as of 5/2022)	41 (11.7)	36 (5.2)*
Caregiver present	8 (67 %)	5 (50 %)
Duration of interview (minutes)		
In-person	85.6 (21.1)	85 (15.2)
Zoom	82.8 (30.3)^‡^	81 (0)

Note: SD = standard deviation.

* One individual later decided not to pursue surgery.

‡ One audio file was corrupted; duration of this interview could not be determined.
