# Supplementary material for: Patient experiences of resection versus responsive neurostimulation for drug-resistant epilepsy
Source: Epilepsy Behav. Author manuscript; Available in PMC 2024 Jun 18. (PMC11185832; doi:10.1016/j.yebeh.2024.109707)
Supplement: Interview Guide [file NIHMS1997126-supplement-Interview_Guide.pdf]

## APPENDIX A: SEMI-STRUCTURED INTERVIEW GUIDE

*At the point of the interview, the respondents have already received a consent form during the first clinical encounter or via email which details the study and research process in detail. Before starting the interview, the researchers paraphrase the most important points of the consent form content once again:*

Thank you very much for agreeing to participate in this interview. As we explained to you when we first met you at the epilepsy center, we are interviewing you to better understand what patients and their caregivers think about experiences with surgical treatments for brain function. It is important to know that there are no right or wrong answers to any of our questions, we are interested in your own experiences and views. Also, your participation in this study is voluntary and your decision to participate, or not participate, will not affect the care you currently receive. You also have the right to withdraw from the study at any point, whether during the interview or afterwards, without penalty or loss of benefits to which you are otherwise entitled.

The interview should take approximately one hour depending on how much information you would like to share with us. And with your permission, we would like to audio record the interview. All responses will be kept confidential. This means that your de-identified interview responses will only be shared with research team members. We will also make sure that any information we include in our articles and reports does not identify you as the respondent. You may decline to answer any question or stop the interview at any time and for any reason. Are there any questions about what we have just explained? May we turn on the digital recorder?

---

### INTERVIEW

#### Beginning of the interview

*Please note that this guide only represents the main themes to be discussed with the participants and as such does not include the various prompts that may also be used (examples given for each question). Non-leading and general prompts will also be used, such as "Can you please tell us a little bit more about that?" and "What does that look like for you".*

**1) Discussion of Observed Visit**

Open the interview by discussing the observed clinic visit.

1.1. Ask the patient/caregiver to explain what happened during that visit in their own words.

1.2. Explore any notable aspects of the visit that made it into the field notes.

1.3. Explore the patient/caregiver relationship with their healthcare providers – not just the physician/neurologist but also the other members of the care team.

1.4. Also explore their feelings about the clinic as a place to get healthcare and how this compares to other places where they receive care.

Use the discussion of the visit to establish rapport and get a sense of the patient/caregiver comfort level discussing

## 2) Personal Background

Explore any issues of personal background

2.1. Where did you grow up?

2.2. Go to school?

2.3. What about brothers or sisters?

2.4. What kind of work have you done in your life?

2.5. Spouses or partners?

2.6. What about children?

Use this portion of the interview to explore and document any previous discussions of what the patient/caregiver considers the patient's authentic self.

## 3) Illness History Get a detailed social history of the illness from the perspective of the patient/caregiver.

Get a detailed social history of the illness from the perspective of the patient/caregiver.

3.1. How was the illness discovered and diagnosed?

3.2. What types of treatments has the patient pursued?

3.3. Were there treatment options that were considered but not pursued?

3.4. What has been happening recently in terms of treatment?

3.5. How has it affected the patient's/caregiver's life?

3.6. Work?

3.7. Relationships with other people?

.3.8 What about past illness experiences?

Be sure to explore the various ups-and-downs in the illness history, particularly if there were times when symptoms impacted day to day quality of life or turning points where management of the illness assumed different intensity.

Ask about significant past illness experiences of family or others.

4) **Fears and Hopes for Medical Care**

Focused discussion of the particular illness condition and health in general.

4.1 Explore what kinds of outcomes the patient/caregiver would consider “successful” management of the illness and why.

4.2. Discuss fears the illness raises and how the patient/caregiver manages or addresses those issues day to day.

4.3. Explore the roles of healthcare providers, family, and other caregivers as they impact hopes of success or fears of illness.

4.4. How are important (and everyday) treatment decisions made?

4.5. Are some types of decisions made more by the patient and others in discussion with caregivers?

4.6. What about the role of providers in decision-making?

Use this section of the interview to explore the dynamics of healthcare decision making.

5) **Practical and Cultural Barriers to Medical Care Access**

Focused discussion of the particular barriers to medical care the patients faced, from first signs of illness to diagnosis and surgical evaluation or intervention.

5.1. Discuss the financial aspects and potential difficulties the patients encountered

5.2. Examine the patients experience with health insurance and the formal health-care system

5.3. Discuss accessibility of diagnostic services, primary care awareness and referral time for surgical evaluation

5.4. Explore geographic barriers the patients experienced, such as transportation, access to neurological care nearby, etc.

5.5. Were there any particular informational and language needs and how were they or weren’t they met?

5.6. Did the patients experience discriminatory treatment based on their race/ethnicity, culture or social status?

Use this section of the interview to explore the various practical and cultural issues that arose over the patient's course of illness and treatment history

6) **Conclusion**

Conclude the interview by inquiring whether there are any other issues that the patient/caregiver would like to discuss that have not yet been raised.

6.1 Is there anything else that you would like to comment on that we haven't already asked you about?

6.2 Are there any questions you would like to ask us?

Thank you very much for your time and the information you shared today.
